# Collective dynamics of actin and microtubule and its crosstalk mediated by FHDC1

**DOI:** 10.3389/fcell.2023.1261117

**Published:** 2024-03-19

**Authors:** Chee San Tong, Maohan Su, He Sun, Xiang Le Chua, Ding Xiong, Su Guo, Ravin Raj, Nicole Wen Pei Ong, Ann Gie Lee, Yansong Miao, Min Wu

**Affiliations:** ^1^ Department of Cell Biology, Yale University School of Medicine, New Haven, CT, United States; ^2^ Department of Biological Sciences, Centre for Bioimaging Sciences, Singapore, Singapore; ^3^ Mechanobiology Institute, National University of Singapore, Singapore, Singapore; ^4^ School of Biological Sciences, Nanyang Technological University, Singapore, Singapore; ^5^ State Key Laboratory of Oral Diseases, National Clinical Research Center for Oral Diseases, West China Hospital of Stomatology, Sichuan University, Chengdu, China; ^6^ Special Programme in Science, National University of Singapore, Singapore, Singapore

**Keywords:** actin waves, microtubule, formins, FHDC1, cell cortex

## Abstract

The coordination between actin and microtubule network is crucial, yet this remains a challenging problem to dissect and our understanding of the underlying mechanisms remains limited. In this study, we used travelling waves in the cell cortex to characterize the collective dynamics of cytoskeletal networks. Our findings show that Cdc42 and F-BAR-dependent actin waves in mast cells are mainly driven by formin-mediated actin polymerization, with the microtubule-binding formin FH2 domain-containing protein 1 (FHDC1) as an early regulator. Knocking down FHDC1 inhibits actin wave formation, and this inhibition require FHDC1’s interaction with both microtubule and actin. The phase of microtubule depolymerization coincides with the nucleation of actin waves and microtubule stabilization inhibit actin waves, leading us to propose that microtubule shrinking and the concurrent release of FHDC1 locally regulate actin nucleation. Lastly, we show that FHDC1 is crucial for multiple cellular processes such as cell division and migration. Our data provided molecular insights into the nucleation mechanisms of actin waves and uncover an antagonistic interplay between microtubule and actin polymerization in their collective dynamics.

## Introduction

Actin filaments and microtubules play integral roles in numerous cellular processes, including cell motility, division, and trafficking. Both actin filaments and microtubules are highly dynamic and undergo continuous growth and shrinkage to facilitate rapid cellular responses to environment cues. In addition, the interaction and coordination between actin and microtubule cytoskeletal networks are crucial for a wide array of cellular activities (Mitchison and Kirschner, 1988; Rodriguez et al., 2003; Akhshi et al., 2014). Significant advancements have been made in understanding the interactions between actin and microtubules through *in vitro* reconstitution (Griffith and Pollard, 1978) and cell-free reconstitution (Sider et al., 1999; Waterman-Storer et al., 2000; Colin et al., 2018) over the past four decades. These reconstitution approaches have been proven valuable in understanding the effects of proteins operating at the interface between actin and microtubule on nucleation (Okada et al., 2010; Henty-Ridilla et al., 2016), stability (Bartolini et al., 2008) and co-organization of these filaments (Sattilaro, 1986; Roger et al., 2004; Preciado López et al., 2014; Elie et al., 2015). Yet, elucidating the cooperative behavior and mutual influence of cytoskeletal components in living cells still present considerable challenges. The complexity arises from facts such as the crowdedness of cytoskeletal network, the complex trajectories and dynamics of the cytoskeleton, and higher-order feedback loops that exist between them in a living system.

Some of the valuable experimental systems used in the literature to elucidate the crosstalk between actin and microtubules in cells are the focal adhesion (Palazzo and Gundersen, 2002; Stehbens and Wittmann, 2012) or the adherent junction (Higashi et al., 2018), both of which provide localized signals that facilitate the analysis. Recently, actin waves have emerged as a common theme of cortical organization in many single cell systems (Roy, 2016; Beta and Kruse, 2017; Devreotes et al., 2017; Inagaki and Katsuno, 2017; Deneke and Di Talia, 2018; Goldbeter, 2018; Wu and Liu, 2021; Beta et al., 2023; Riedl and Sixt, 2023). These waves play important roles in signal transduction (Wu et al., 2013, 2018; Xiong et al., 2016; Hörning et al., 2021; Tong et al., 2023), cell division (Bement et al., 2015; Xiao et al., 2017; Dimitracopoulos et al., 2020; Flemming et al., 2020; Moore et al., 2021; Michaud et al., 2022), cellular morphogenesis (Miao et al., 2019; Bhattacharya et al., 2020; Wigbers et al., 2021; Yao et al., 2022), cell polarity (Arai et al., 2010; Taniguchi et al., 2013; Banerjee et al., 2022), single cell migration and collective migration (Gerisch et al., 2004; Weiner et al., 2007; Miao et al., 2017; Bolado-Carrancio et al., 2020; Zhan et al., 2020; Ecker and Kruse, 2021; Banerjee et al., 2022; Riedl et al., 2023). Collective dynamics of cytoskeletal components not only amplify weaker signals originating from dynamics of individual filament, but also serve as a readout for higher-order feedback in the system that only exist with groups of filaments (Yang and Wu, 2018). In particular, the temporal information from oscillatory behaviors is ideal for studying the crosstalk and feedback mechanisms of intertwined molecular networks (Chua et al., 2023).

In this paper, we used oscillatory travelling waves on the cell cortex to examine the collective dynamics of actin assembly and the interplay between cytoskeletal networks. We have previously found the involvement of Cdc42 and its effector FBP17 in mediating actin waves on the cortex of mast cells (Wu et al., 2013, 2018; Xiong et al., 2016). Here we focused on identifying key regulators important for nucleating actin in these waves. We specifically explored the role of formins and microtubules. Our results revealed that multiple formins including Formin-like protein 1 (FMNL1), mDia3 (also called Protein diaphanous homolog 2/DIAPH2 or Diaphanous-related Formin 2/DRF2), and FH2 Domain Containing 1 (FHDC1) (also called Inverted Formin 1/INF1) are involved in the early phase of actin wave. Among them, FHDC1 is the only formin that is localized on microtubules. Depletion of FHDC1 inhibited actin wave formation. Interestingly, collective shrinking of microtubules coincides with actin wave nucleation. In addition, stabilizing microtubule with taxol inhibits actin wave, suggesting an antagonistic relationship between microtubule and actin polymerization in the context of wave dynamics. Finally, in the absence of FHDC1, cells showed defects in cell division, altered cell morphology and impaired locomotion. Collectively, our observations underscore the critical role of formins in actin wave nucleation and FHDC1 in mediating the crosstalk between microtubule and actin network in travelling waves.

## Results

### Formins are major nucleators of actin in cortical waves

**FIGURE 1:**
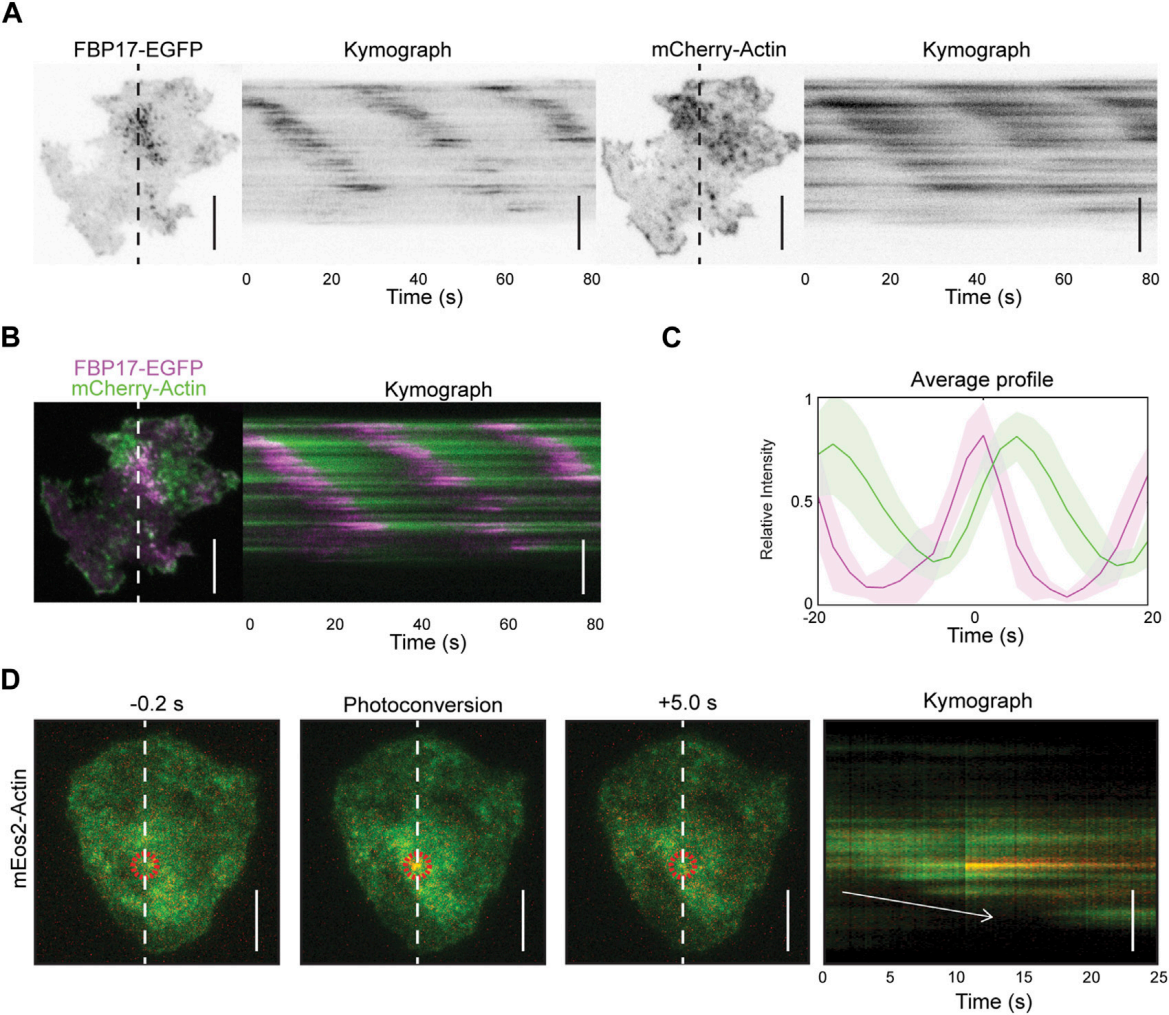
Actin waves propagation is due to *de novo* nucleation of actin. **(A)** Representative TIRFM micrographs and kymograph of a cell co-transfected with FBP17-EGFP and mCherry-Actin or LifeAct-mRFPruby (*n* = 14 cells; 3 independent experiments). Grayscale micrographs and kymographs are displayed with an inverted lookup table. Scale bars: 10 μm. **(B)** Two-color merge of TIRFM micrograph and kymograph of a representative cell co-transfected with FBP17-EGFP (magenta) and mCherry-Actin (green) from Panels **(A)**. Scale bar: 10 μm. **(C)** Representative average profile of mCherry-Actin (green) aligned with respect to FBP17-EGFP (magenta). The solid lines represent the mean intensities, and the shaded region represent the standard deviations of the intensities. **(D)** Representative TIRFM micrographs and kymograph of cells transfected with mEos2-actin before and after photoconversion (*n* = 98 cells; 12 independent experiments). The mEos2-actin punctum was photoconverted in the region indicated by the red circle. White arrow indicates direction of wave propagation. Scale bars: 10 μm.

In our previous studies, we have reported that resting and stimulated tumor mast cells (rat basophilic leukemia cells, or RBL cells) display cortical travelling waves of FBP17 and actin (Wu et al., 2013). Using total internal reflection fluorescence microscopy (TIRFM) at a fast acquisition rate (5 Hz), we observed waves of FBP17 and actin, with actin trailing behind FBP17 (Figures 1A–C, Supplementary Movie S1). To visualize the turnover of individual actin puncta, we expressed mEos2-actin, a fluorescent protein that undergoes irreversible photo-conversion, shifting its emission peak from green (516 nm) to red (581 nm) (Mckinney et al., 2009), and selectively illuminated a single punctum in the mEos2-actin wave. We observed that the activated mEos2-actin remained in the same spot without any apparent spatial shift as actin waves propagate (Figure 1D), similar to what we previously reported for FBP17 clusters (Wu et al., 2018). Thus, actin waves unlikely propagate by advection, rather, waves propagate through *de novo* nucleation of actin at the wave front, similar to the working model of actin waves in other systems (Bretschneider et al., 2009; Bement et al., 2015).

**FIGURE 2:**
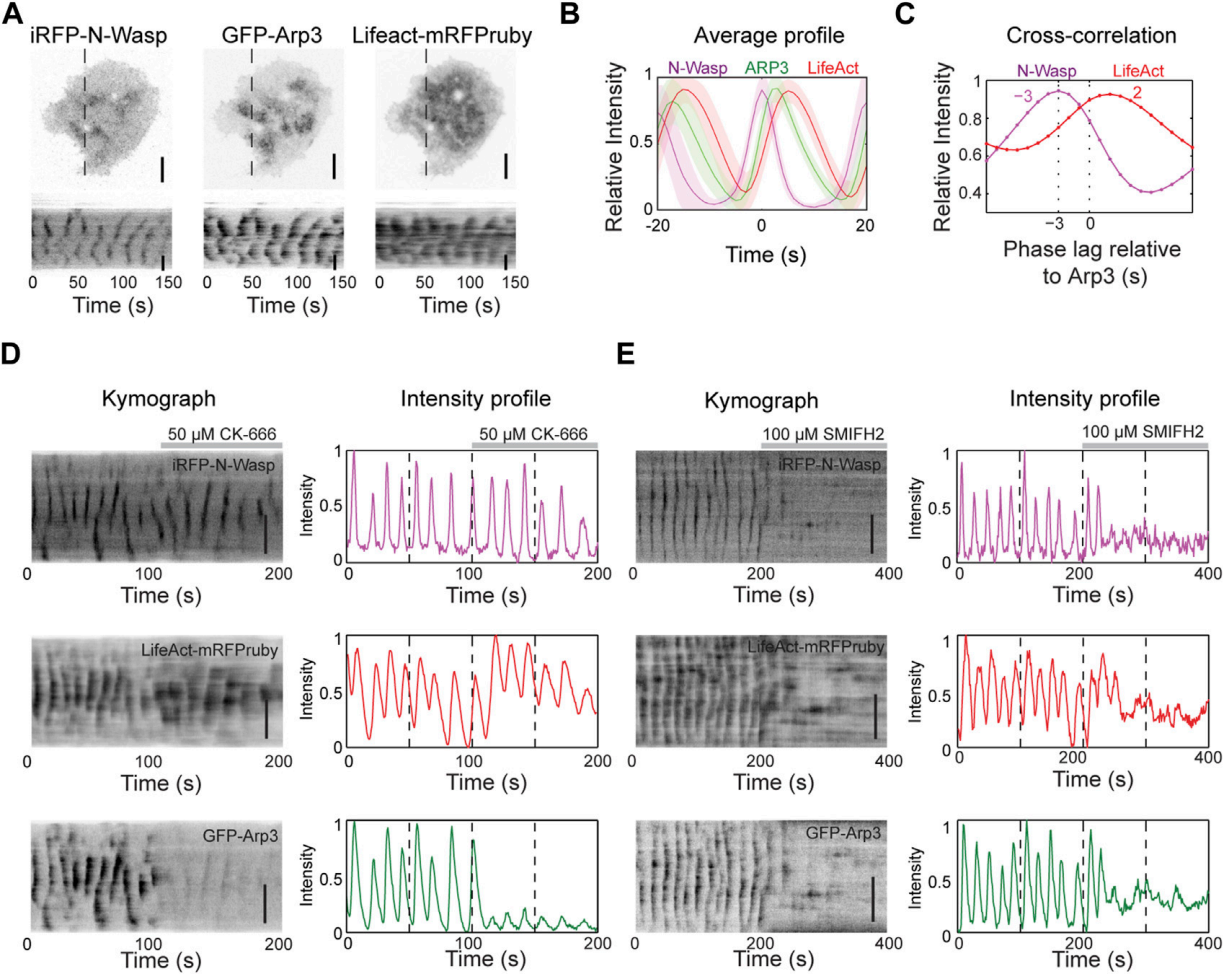
Actin nucleation in the waves is primarily mediated by formins. **(A)** Representative TIRM micrographs and kymographs of a cell co-transfected with iRFP-N-Wasp, GFP-Arp3 and LifeAct-mRFPruby (*n* = 74 cells; 7 independent experiments). Grayscale micrographs and kymographs are displayed with an inverted lookup table. Scale bars: 10 μm. **(B)** Representative average profile of GFP-Arp3 (green) and LifeAct-mRFPruby (red) aligned with respect to iRFP-N-Wasp (magenta) (*n* = 74 cells; 7 independent experiments). The solid lines represent the mean intensities, and the shaded regions represent the standard deviations of the intensities. **(C)** Representative cross-correlation analysis of the time differences between the peak intensities of iRFP-N-Wasp (magenta) and LifeAct-mRFPruby (red) relative to GFP-Arp3. **(D)** Representative kymographs and intensity profiles of a cell co-transfected with iRFP-N-Wasp (magenta), LifeAct-mRFPruby (red) and GFP-Arp3 (green), and treated with 50 µM CK-666 (*n* = 8 cells; 2 independent experiments). Grayscale kymographs are shown with inverted lookup table. Scale bars: 10 μm. **(E)** Representative kymographs and intensity profiles of a cell co-transfected with iRFP-N-Wasp (magenta), LifeAct-mRFPruby (red) and GFP-Arp3 (green), and treated with 100 µM SMIFH2 (*n* = 23 cells; 6 independent experiments). Grayscale kymographs are shown with inverted lookup table. Scale bars: 10 μm.

To understand the nucleation mechanisms involved in actin dynamics during wave propagation, we first tested Arp2/3 complex, a major actin nucleator. We expressed iRFP-N-Wasp (an Arp2/3 activator), GFP-Arp3 (a component of the Arp2/3 complex) and LifeAct-mRFPruby (F-actin marker) and visualized their cortical dynamics. Our results show that N-Wasp and Arp3 exhibit similar patterns of cortical waves as actin (Figures 2A, B). To quantify the temporal relationship between these proteins, we performed cross-correlation analysis on their intensity profile over time (Figure 2C). The analysis revealed that Arp3 puncta lagged the corresponding N-Wasp puncta by approximately 3 s but preceded F-actin by approximately 2 s (Figure 2C).

To determine the necessity of the Arp2/3 complex in the nucleation of actin waves, we used a chemical inhibitor of Arp2/3 CK-666. CK-666 keeps the Arp2/3 complex in an inactive state, preventing it from nucleating new actin filaments at the edges of existing filaments (Hetrick et al., 2013). Surprisingly, despite a significant decrease in the intensity and dynamics of Arp3 waves after the addition of 50 μM CK-666, we observed that N-Wasp and actin wave propagation persisted (Figure 2D). In addition, there was a slight increase in LifeAct intensity (Figure 2D), indicating that Arp2/3 may not be responsible for the bulk of F-actin in actin waves. These results motivated us to explore the involvement of formins in nucleating actin waves. We treated cells with a broad range formin inhibitor SMIFH2, which impedes the FH2 domains of formins and reduces their affinity for the barbed ends of actin filaments (Rizvi et al., 2009). Remarkably, both N-Wasp and actin wave formations were completely abolished (Figure 2E). SMIFH2 has been shown to have a non-specific effect on myosinII at concentrations exceeding 30 µM (Alieva et al., 2019; Nishimura et al., 2021). With this potential caveat, these pharmacological experiments are consistent with the possibility that formins are the major contributors to the nucleation Cdc42/FBP17-dependent actin waves in mast cells.

### FHDC1 is an early-phase regulator of cortical waves

**FIGURE 3:**
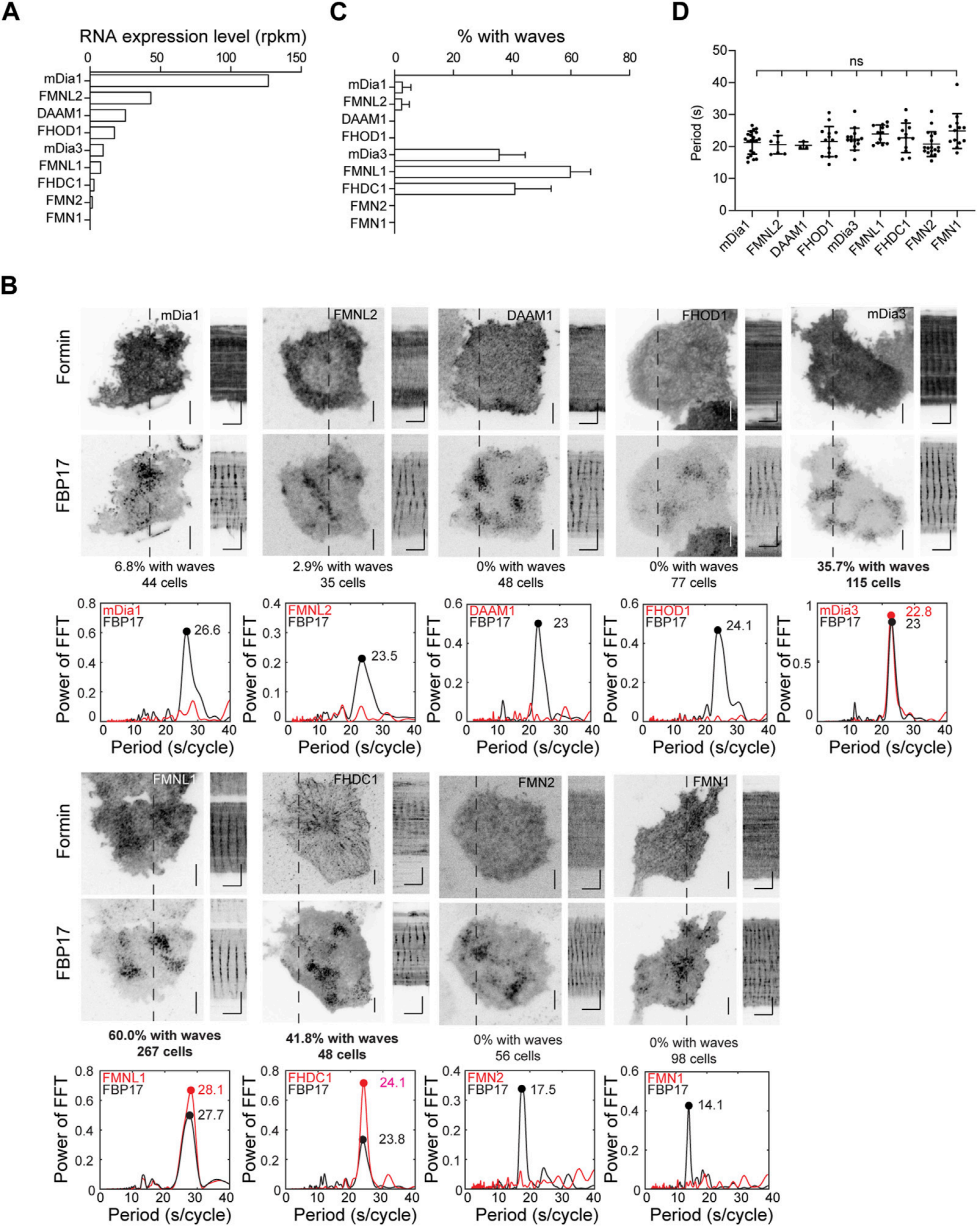
Localization of formins in the cortical travelling waves. **(A)** Formin transcripts expression levels in RBL cells determined by RNA-Seq. Transcript expression levels were quantified as reads per kilobase per million mapped reads (RPKM). **(B)** Representative micrographs and kymographs of formin waves imaged over 2 min, along with FBP17 as wave marker. Grayscale micrographs and kymographs are displayed with an inverted lookup table. Horizontal scale bars: 1 min. Vertical scale bars: 10 μm. Representative FFT analyses of formin waves. The three formins with the highest populations of cells displaying formin waves are highlighted in bold. **(C)** Quantification of the percentage of cells with formin travelling waves (4 – 23 independent experiments for each formin). **(D)** Quantification of frequency distributions of cortical FBP17 waves in cells overexpressing various formins. Error bars represent the standard error of mean (SEM).

To determine which formins are involved in actin wave formation, we examined the transcript expression levels of various formins in RBL cells using RNA-seq analysis (Figure 3A). Nine formins were expressed at varying levels, including Diaphanous Related Formin 1 (mDia1 or DIAPH1), mDia3, Disheveled-Associated Activator of Morphogenesis 1 (DAAM1), FMNL1, Formin Homology 2 Domain Containing 1 (FHOD1), Formin like 2 (FMNL2), Formin 1 (FMN1), Formin 2 (FMN2) and FHDC1. We proceeded to express each of these formins and visualize their cortical localizations, using FBP17 as a wave marker. We observed varying percentages of cells showing waves of different formins. To eliminate any bias in the visual selection process of cells positive for waves, we performed fast Fourier transformations (FFTs) on the intensity profiles, using the presence of single and distinct FFT peaks in the period of 0–40 s range as a criterion for robust oscillations. Based on this criterion, FMNL1, FHDC1 and mDia3 displayed oscillatory wave patterns in 60.0%, 41.8% and 35.7% of the cells with FBP17 waves, respectively. For the rest of the formins, we either did not observe wave patterns or they only appeared in rare cells (Figure 3B). The percentage of cells exhibiting formin waves did not correlate with the individual transcript levels of the formins determined by RNA-seq analysis (Figure 3C), suggesting that the differences observed in wave participation reflect selective involvement in the pattern. Lastly, we also quantified the distribution of FBP17 frequencies when different formins were overexpressed and found no significant differences (Figure 3D).

**FIGURE 4:**
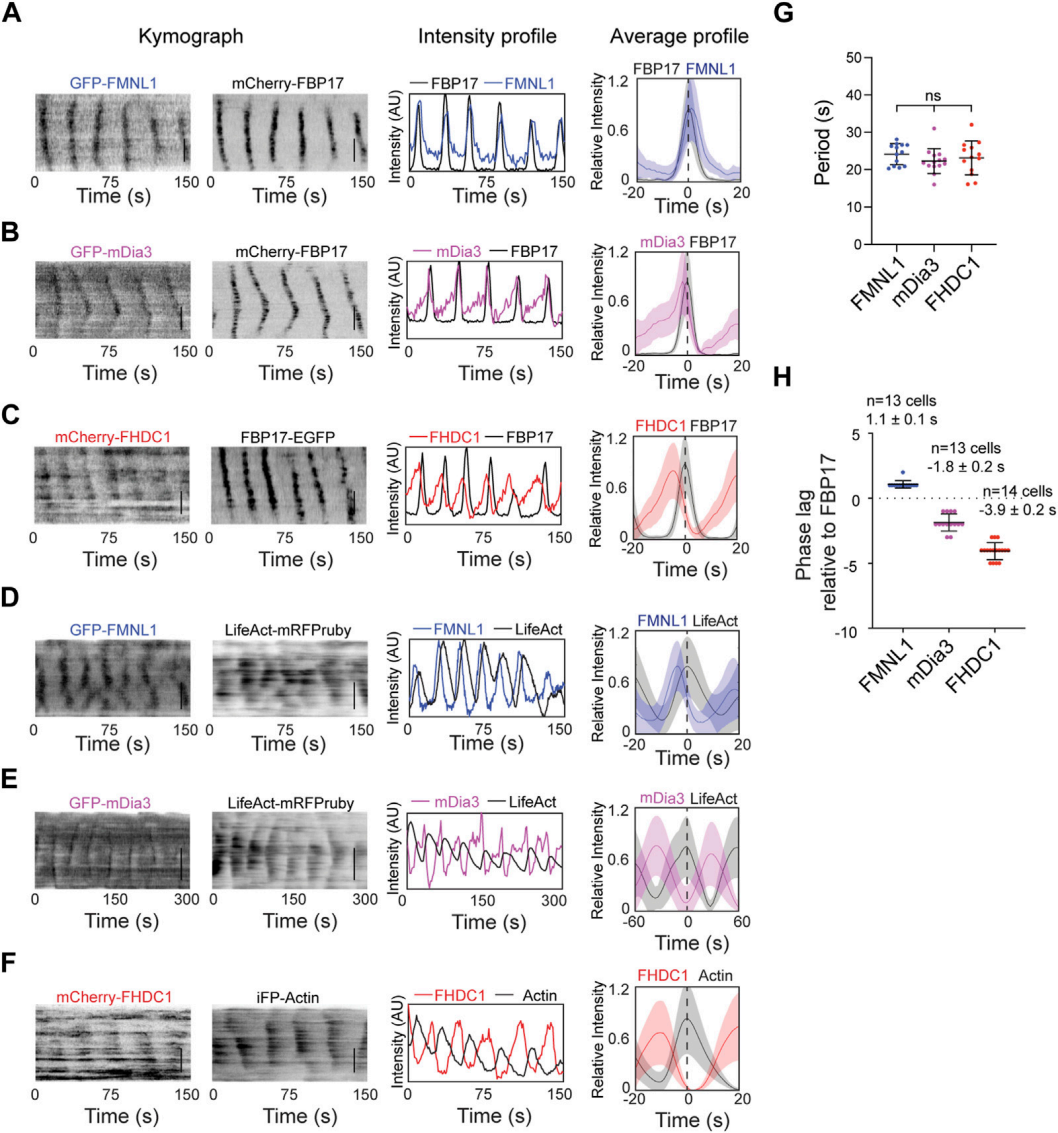
FHDC1 is an early phase regulator of cortical actin waves. **(A)** Representative kymographs, intensity profile and average profile of a cell co-transfected with GFP-FMNL1 (blue) and mCherry-FBP17 (black) captured by TIRFM. Average profile of GFP-FMNL1 (blue) is aligned with respect to mCherry-FBP17 (black). The solid lines represent the mean intensities, and the shaded regions represent the standard deviations of the intensities. Dashed line represents time when the intensity of FBP17 peaks. Grayscale kymographs are shown with inverted lookup table. Scale bars: 10 μm. **(B)** Representative kymographs, intensity profile and average profile of a cell co-transfected with GFP-mDia3 (magenta) and mCherry-FBP17 (black) captured by TIRFM. Average profile of GFP-mDia3 (magenta) is aligned with respect to mCherry-FBP17 (black). Dashed line represents time when the intensity of FBP17 peaks. Grayscale kymographs are shown with inverted lookup table. Scale bars: 10 μm. **(C)** Representative kymographs, intensity profile and average profile of a cell co-transfected with mCherry-FHDC1 (red) and FBP17-EGFP (black) captured by TIRFM. Average profile of mCherry-FHDC1 (red) is aligned with respect to FBP17-EGFP (black). The solid lines represent the mean intensities, and the shaded regions represent the standard deviations of the intensities. Dashed line represents time when the intensity of FBP17 peaks. Grayscale kymographs are shown with inverted lookup table. Scale bars: 10 μm. **(D)** Representative kymographs, intensity profile and average profile of a cell co-transfected with GFP-FMNL1 (blue) and LifeAct-mRFPruby (black) captured by TIRFM. Average profile of GFP-FMNL1 (blue) is aligned with respect to LifeAct-mRFPruby (black). The solid lines represent the mean intensities, and the shaded regions represent the standard deviations of the intensities. Dashed line represents time when the intensity of LifeAct peaks. Grayscale kymographs are shown with inverted lookup table. Scale bars: 10 μm. **(E)** Representative kymographs, intensity profile and average profile of a cell co-transfected with GFP-mDia3 (magenta)and LifeAct-mRFPruby (black) captured by TIRFM. Average profile of GFP-mDia3 (magenta) is aligned with respect to LifeAct-mRFPruby (black). The solid lines represent the mean intensities, and the shaded regions represent the standard deviations of the intensities. Dashed line represents time when the intensity of LifeAct peaks. Grayscale kymographs are shown with inverted lookup table. Scale bars: 10 μm. **(F)** Representative kymographs, intensity profile and average profile of a cell co-transfected with mCherry-FHDC1 (red) and GFP-Actin (black) captured by TIRFM. Average profile of mCherry-FHDC1 (red) is aligned with respect to GFP-Actin (black). The solid lines represent the mean intensities, and the shaded regions represent the standard deviations of the intensities. Dashed line represents time when the intensity of Actin peaks. Grayscale kymographs are shown with inverted lookup table. Scale bars: 10 μm. **(G)** Quantification of frequency distributions of cortical FMNL1, mDia3 and FHDC1 waves in cells. Error bars represent the standard error of mean (SEM). **(H)** Quantification of the phase lag of FMNL1, FHDC1 and mDia3 with respect to FBP17. Error bars represent the standard error of mean (SEM).

Based on their high probabilities of recruitment in the cortical waves, we further characterized FMNL1, mDia3 and FHDC1 in actin waves. They showed differences in their phases of recruitment. GFP-FMNL1 and mCherry-FBP17 showed strong co-localization. FMNL1 intensity oscillations overlapped extensively with FBP17 in phase (Figure 4A). In contrast, mDia3 and FHDC1 peaked before FBP17 (Figures 4B, C). Waveforms for these formins also differed. The assembly of FHDC1 and mDia3 on the membrane occurred at a slower rate compared to their disassembly, as indicated by gentle rising phases and sharp falling phases (Figures 4B, C). All three formins preceded actin in wave (Figures 4D–F). Despite their differences, in phases of recruitment and waveforms, FMNL1, mDia3 and FHDC1 all shared the same propagation periodicities (Figure 4G). On average, FMNL1 peaks approximately 1 s later than FBP17 (1.1 ± 0.1 s, 10 cells), while FHDC1 waves precede FBP17 by approximately 4 s (−3.9 ± 0.2 s, 13 cells), and mDia3 waves preceded FBP17 by approximately 2 s (−1.8 ± 0.2 s, 14 cells) (Figure 4H). Taken together, these data suggest that FHDC1 and mDia3 likely act as early phase regulators of actin wave formation.

### Interactions of FHDC1 with both actin and microtubule regulate wave propagation

**FIGURE 5:**
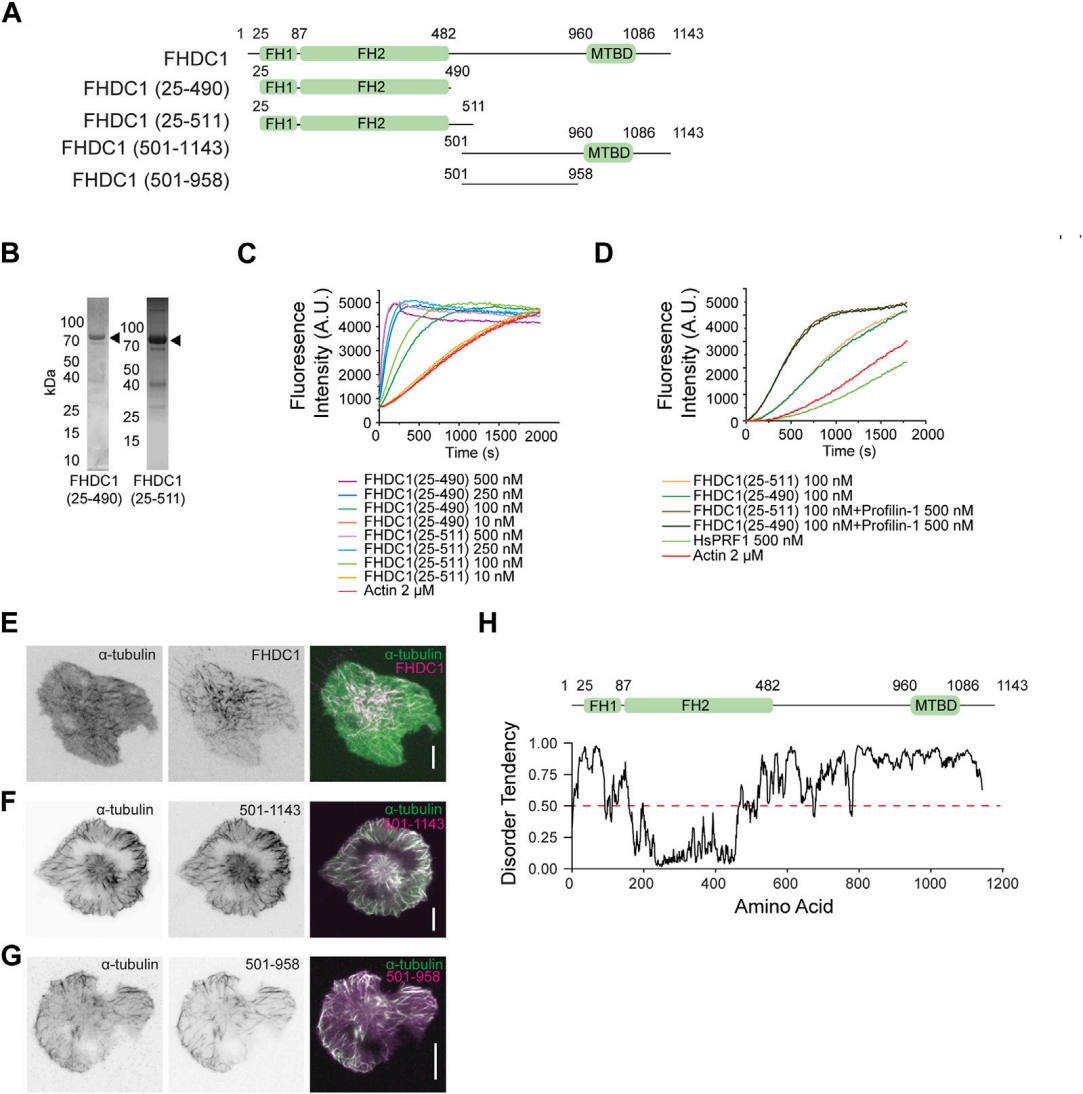
The FH domains of FHDC1 are sufficient for actin polymerization. **(A)** Domain organization of wild type and truncated mutant constructs of FHDC1. **(B)** Purified FHDC1 truncating variants analyzed using Coomassie blue-stained SDS-PAGE gels. **(C)** Pyrene-actin polymerization assay performed using 2 μM actin (5% pyrene-labeled) in the presence of the indicated concentration of FHDC1(25-490) and FHDC1(25-511) truncated variants. **(D)** Pyrene-actin polymerization assay using 2 μM actin (5% pyrene-labeled) in the presence of the 100 nM FHDC1(25-490), 100 nM FHDC1(25-511) and 500 nM Profilin-1. **(E)** Representative micrographs of a cell co-transfected with GFP-α-tubulin and mCherry-FHDC1 captured by TIRFM. Grayscale micrographs and are shown with inverted lookup table. Scale bar: 10 μm. Two-color merge of TIRFM micrograph is represented by the following pseudocolors: GFP-α-tubulin (green), mCherry-FHDC1 (magenta). **(F)** Representative micrograph of a cell co-transfected with mCherry-α-tubulin and GFP-FHDC1 (501-1143) captured by TIRFM. Grayscale micrographs and are shown with inverted lookup table. Scale bar: 10 μm. Two-color merge of TIRFM micrograph is represented by the following pseudocolors: mCherry-α-tubulin (green), GFP-FHDC1 (501-1143) (magenta). **(G)** Representative micrograph of a cell co-transfected with mCherry-α-tubulin and GFP-FHDC1 (501-958) captured by TIRFM. Grayscale micrographs and are shown with inverted lookup table. Scale bar: 10 μm. Two-color merge of TIRFM micrograph is represented by the following pseudocolors: mCherry-α-tubulin (green), GFP-FHDC1 (501-958) (magenta). **(H)** Diagram illustrating the prediction of intrinsically disorder region (IDR) in the full-length FHDC1 using the IUPRED2 algorithm (https://iupred2a.elte.hu/). Regions with an IDR score above 0.5 are considered to be intrinsically disordered.

We focused on investigating FHDC1 in our study due to its early recruitment to cortical waves and distinct filamentous localization pattern, which distinguishes it from the other formins. Unlike typical formins that have conserved formin homology (FH) domains at their C-terminal, FHDC1 exhibits an inverted arrangement with its FH1 and FH2 domains in the N-terminal half (Figure 5A). To test whether the FH domains could polymerize actin, we purified two truncation mutants of FHDC1, namely FHDC1 (25-490) and FHDC1 (25-511) (Figure 5B), and performed *in vitro* pyrene actin polymerization assays. The results confirmed that both proteins were capable of nucleating actin polymerization (Figure 5C). Moreover, the nucleation activity could be accelerated by Profilin-1 (Figure 5D), a known regulator of actin dynamics.

Since we observed that the full-length FHDC1 co-localized with α-tubulin (Figure 5E, Supplementary Movie S2), we further characterized the relationship between FHDC1 and microtubules. We generated truncated constructs encompassing different regions of FHDC1 and examined their co-localization with α-tubulin. FHDC1 truncated mutant lacking FH domains (FHDC1 501-1143) also localized to microtubules (Figure 5F). The C-terminus of FHDC1 was previously reported to contain a microtubule-binding domain (FHDC1 958-1143) (Young et al., 2008). However, the central region lacking the C-terminus domain (FHDC1 501-958) was found to be sufficient for microtubule association (Figure 5G). This region has not been previously characterized, and it is predicted to be intrinsically disordered (Figure 5H). These findings confirmed the association of microtubule with FHDC1 and suggest that the region responsible for FHDC1’s binding to microtubule extends beyond the previously identified microtubule-binding domain.

**FIGURE 6:**
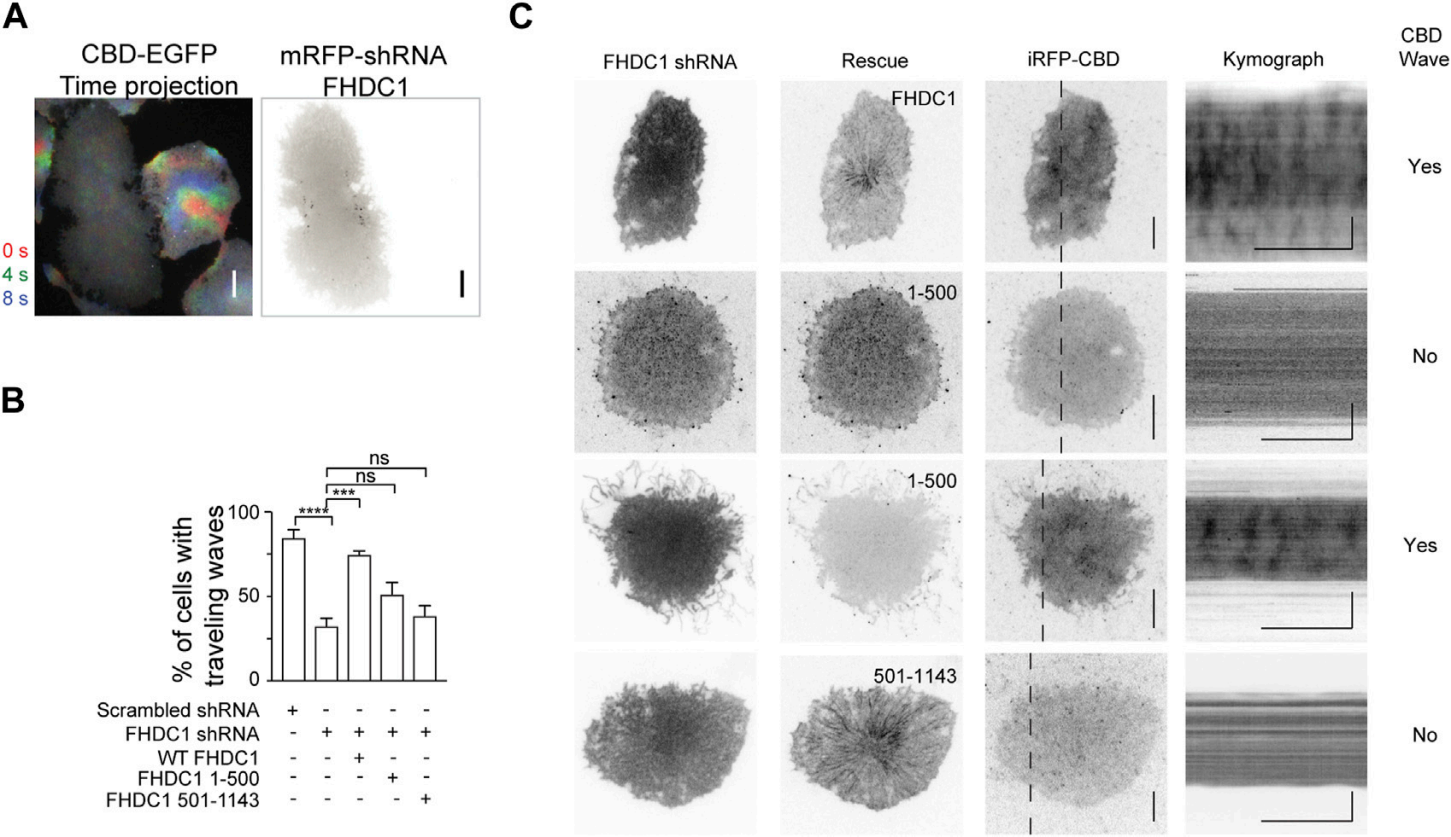
Both actin and microtubule interacting domains of FHDC1 are required for wave propagation. **(A)** Representative time projection image of CBD wave propagation over 8 s (*n* = 242 cells; 8 independent experiments). The pseudocolors representing each frame are as follows: Red: 0 s, Green: + 4 s. Blue: + 8 s. Co-expression of FHDC1 shRNA constructs in an EGFP-CBD stable cell line abolished propagation of CBD wave. Grayscale micrographs are shown with inverted lookup table. Scale bar: 10 μm. **(B)** Quantification of the percentage of cells with CBD travelling waves following transfection with FHDC1 shRNA (*n* = 242 cells; 8 independent experiments) or scrambled shRNA (*n* = 183 cells; 4 independent experiments), compared with rescue by full length GFP-FHDC1 (*n* = 108 cells; 3 independent experiments), GFP-FHDC1 (1-500) (*n* = 92 cells; 3 independent experiments) or GFP-FHDC1 (501-1143) (*n* = 86 cells; 3 independent experiments). **(C)** Representative micrographs and kymographs of cells co-transfected with pRFP-C-RS-FHDC1 shRNA constructs, iRFP-CBD and either full length GFP-FHDC1, GFP- FHDC1 (1-500) or GFP-FHDC1 (501-1143). Grayscale micrographs and kymographs are shown with inverted lookup table. Horizontal scale bars: 1 min. Vertical scale bars: 10 μm.

To test the importance of FHDC1’s various interactions including microtubule association in regulating wave propagation, we attempted to knockdown FHDC1, then rescue the effects of FHDC1 knockdown on wave propagation by re-introducing an shRNA resistant FHDC1 construct of domain-truncation mutants. First, to determine whether FHDC1 is required for wave formation, we performed a knockdown targeting FHDC1 (Figure 6A). The knockdown resulted in a significant reduction in the percentage of cells displaying waves as marked by EGFP-CBD (Cdc42-binding-domain), from 84.8% ± 4.6% to 32.6% ± 4.6% (Scrambled shRNA: *n* = 183 cells; 4 independent experiments; FHDC1 knockdown: *n* = 273 cells; 9 independent experiments, *p* < 0.0001, one-way ANOVA; adjusted *p* < 0.0001, Sidak’s multiple comparison *post hoc* test) (Figure 6B), suggesting that FHDC1 plays an important role in facilitating wave propagation. The re-introduction of full length FHDC1 successfully restored wave propagation to levels observed prior to the knockdown (74.8% ± 2.1%, n = 108 cells; 3 independent experiments, *p* < 0.0001, one-way ANOVA; adjusted *p* = 0.0002, Sidak’s multiple comparison *post hoc* test), confirming the importance of FHDC1 in facilitating wave propagation (Figures 6B, C). We next attempted to rescue with two truncated mutants, FHDC1 (1-500), which only carries the FH domains, and FHDC1 (501-1143), which carries the microtubule binding domain but no actin nucleation domain. FHDC1 (1-500) failed to restore wave propagation (51.3% ± 7.0%, *n* = 92 cells; 3 independent experiments, *p* < 0.0001, one-way ANOVA; adjusted *p* = 0.1271, Sidak’s multiple comparison *post hoc* test) (Figures 6B, C). FH domain showed competence in the *in vitro* actin nucleation assay but FHDC1 (1-500) appears largely diffusive in cells, except that it is localized at tip of filopodia. Thus, these results suggest the microtubule-binding regions are still essential for the correct localization of FHDC1 and its effect on cortical waves. The microtubule-binding segments of FHDC1 (FHDC1 (501-1143)) completely fail to rescue wave propagation in knockdown cells (38.7% ± 5.9% cells, *n* = 86 cells, 3 independent experiments, *p* < 0.0001, one-way ANOVA; adjusted *p* = 0.9448, Sidak’s multiple comparison *post hoc* test) (Figures 6B, C). While statistically insignificant, we note that the percentage of wave propagation is higher when FHDC1 (1-500) is introduced compared to FHDC1 (501-1143), potentially suggesting that FH domain in the absence of microtubule-binding regions could have some activity. These data collectively indicate that both the FH domains and microtubule-binding domains of FHDC1 are important in mediating wave propagation.

### Microtubule depolymerization regulates actin waves

**FIGURE 7:**
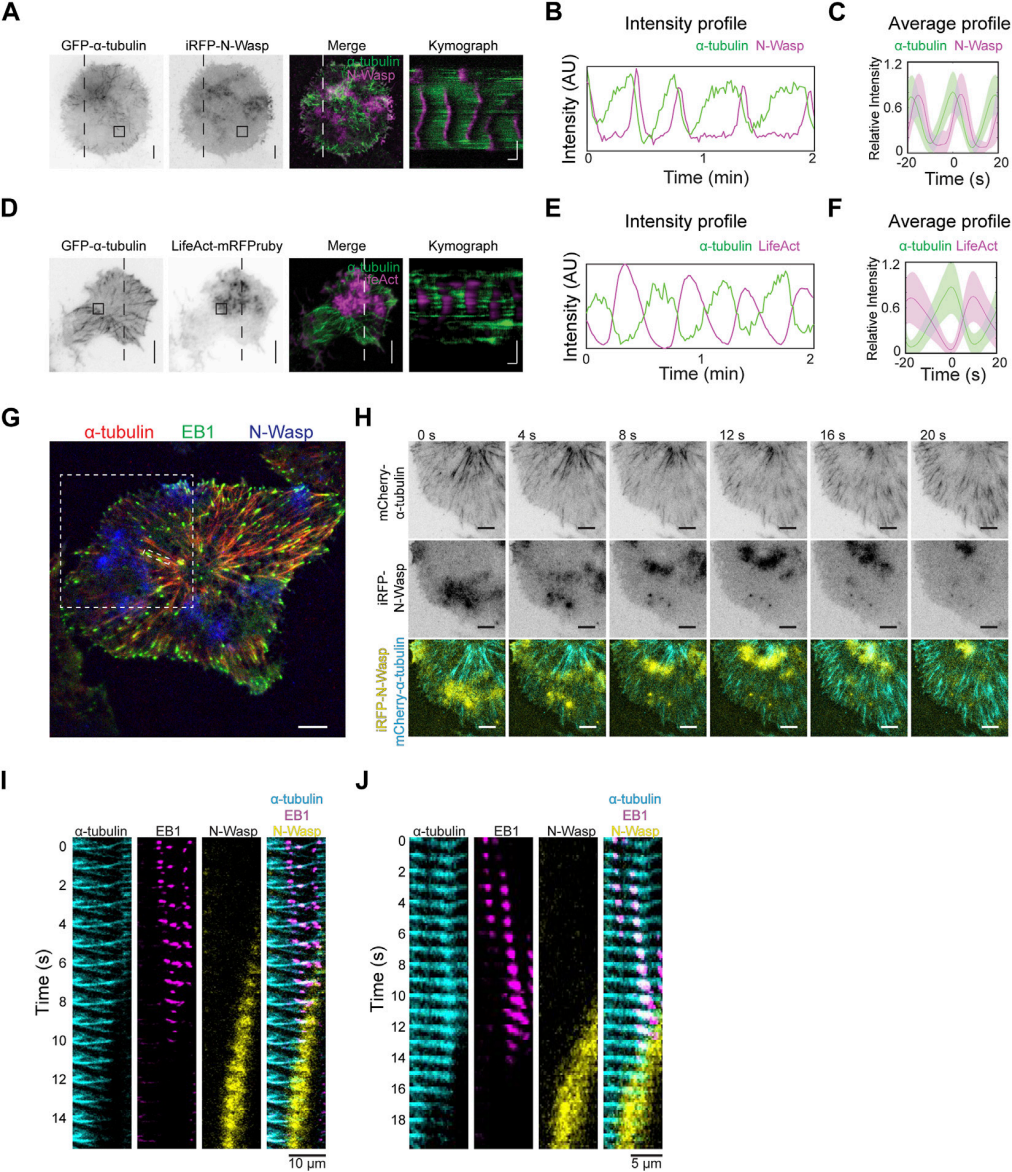
Microtubule depolymerization coincides with actin waves. **(A)** Representative TIRFM micrographs and kymograph of a cell co-transfected with iRFP-N-Wasp and GFP-α-tubulin (30 cells). Grayscale micrographs are shown with inverted lookup table. Horizontal scale bar: 10 s. Vertical scale bars: 10 μm. The box indicates the region-of-interest used to plot the intensity profile in panel **(B)**. Two-color merge of TIRFM micrograph and kymograph are represented by the following pseudocolors: GFP-α-tubulin (green), iRFP-N-Wasp (magenta). **(B)** Intensity profile of GFP-α-tubulin (green) and iRFP-N-Wasp (magenta) wave propagation plotted at region marked by the box in Panel **(A)**. **(C)** Representative average profile of iRFP-N-Wasp (magenta) aligned with respect to GFP-α-tubulin (green). The solid lines represent the mean intensities, and the shaded regions represent the standard deviations of the intensities. **(D)** Representative TIRFM micrographs and kymograph of a cell co-transfected with GFP-α-tubulin and LifeAct-mRFPruby (12 cells). Grayscale micrographs are shown with inverted lookup table. Horizontal scale bar: 10 s. Scale bars: 10 μm. The box indicates the region-of-interest used to plot the intensity profile in Panel **(E)**. Two-color merge of TIRFM micrograph and kymograph are represented by the following pseudocolors: GFP-α-tubulin (green), LifeAct-mRFPruby (magenta). **(E)** Intensity profile of GFP-α-tubulin (green) and LifeAct-mRFPruby (magenta) wave propagation plotted at selected region marked by the box in Panel **(D)**. **(F)** Representative average profile of LifeAct-mRFPruby (magenta) aligned with respect to GFP-α-tubulin (green). The solid lines represent the mean intensities, and the shaded regions represent the standard deviations of the intensities. **(G)** Representative TIRFM micrograph of a cell co-transfected with GFP-EB1 (green), mCherry-α-tubulin (red) and iRFP-N-Wasp (blue). White dotted boxes represent regions of interest expanded for montage of coordinated shrinking of a cluster of microtubules filaments in panel H and montage of single microtubule filament shrinkage in Panel **(J)**. Scale bar: 10 μm. **(H)** Grayscale montage of α-tubulin and iRFP-N-Wasp are shown with inverted lookup table. Merged montage of a coordinated shrinking of a cluster of microtubules filaments are displayed with the following pseudocolors: mCherry-α-tubulin (cyan) and iRFP-N-Wasp (yellow). Scale bar: 10 μm. **(I)** Merged montage of multiple microtubule filament shrinkage displayed with the following pseudocolors: mCherry-α-tubulin (cyan), GFP-EB1 (magenta) and iRFP-N-Wasp (yellow). Scale bar: 10 μm. **(J)** Merged montage of a single microtubule filament shrinkage displayed with the following pseudocolors: mCherry-α-tubulin (cyan), GFP-EB1 (magenta) and iRFP-N-Wasp (yellow). Scale bar: 5 μm.

We proceeded to investigate how the dynamics of tubulin waves could be linked to actin waves. Co-imaging GFP-α-tubulin with iRFP-N-Wasp revealed that the disappearance of α-tubulin coincided with the appearance of N-Wasp waves (Figures 7A–C, Supplementary Movie S3). Similarly, when LifeAct-mRFPruby was co-imaged with GFP-α-tubulin, actin waves were observed to trail behind α-tubulin waves (Figures 7D–F). While there was some degree of overlap between the signals of N-Wasp and microtubules (Figures 7B, C), the peaks of actin were found to be anti-phased with microtubule waves (Figures 7E, F). To confirm that disappearance of the microtubule was indeed due to depolymerization rather than leaving the TIRF field, we visualized the dynamics of microtubules tips by co-expressing GFP-EB1 with mCherry-α-tubulin and iRFP-N-Wasp (Figure 7G). We found that the arrival of N-Wasp coincided with the collective shrinkage of microtubule which were tagged with EB1 (Figures 7H–J, Supplementary Movie S4).

**FIGURE 8:**
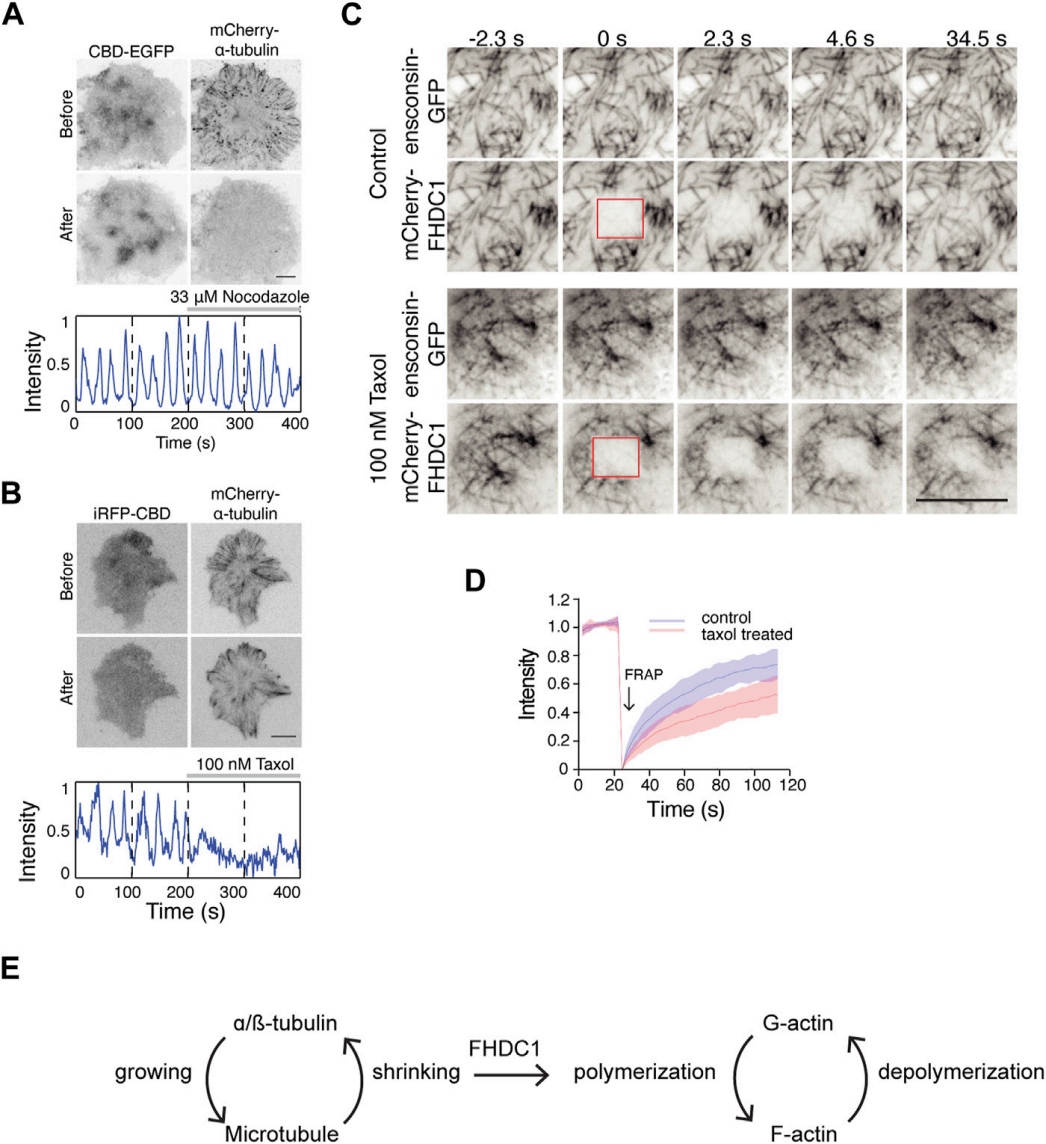
Depolymerization of microtubule regulate cortical waves through FHDC1 **(A)** Top: Representative micrographs of cells stably expressing CBD-EGFP co-transfected with mCherry-α-tubulin before and after 33 μM nocodazole treatment (*n* = 13 cells; 5 independent experiments). Bottom: Intensity profile of CBD-EGFP of the above cell before and after nocodazole treatment over 400 s. Nocodazole treatment is indicated by the gray bar above the intensity profile. **(B)** Top: Representative micrographs of cells co-transfected with iRFP-CBD and GFP-α-tubulin before and after 100 nM taxol treatment (*n* = 5 cells; 3 independent experiments). Bottom: Intensity profile of CBD-EGFP of the above cell before and after taxol treatment over 400 s. Taxol treatment is indicated by the gray bar above the intensity profile. **(C)** Representative micrographs of mCherry-FHDC1 with ensconsin-GFP (microtubule marker) in 100 nM taxol medium or control medium. The photobleached area by 561 nm laser is shown by red boxes. Scale bar: 10 μm. **(D)** Intensity profile of fluorescence recovery after photobleaching (FRAP) of mCherry-FHDC1 in 8 cells for each treatment. Shaded region denotes standard deviation. **(E)** Model of crosstalk between growing and shrinking microtubule and actin regulation by FHDC1.

Next, we examined the importance of microtubule dynamics in regulating actin waves. Preventing microtubule polymerization using 33 µM nocodazole did not affect wave dynamics (Figure 8A). However, stabilizing microtubules with 100 nM taxol resulted in an instant inhibition of wave propagation (Figure 8B). Interestingly, in cells with taxol-stabilized microtubules, the recovery of mCherry-FHDC1 fluorescence after photo-bleaching was significantly reduced compared to untreated cells (Figures 8C, D). Taken together, these results suggest that the dynamic instability of microtubule, rather than microtubule polymerization *per se*, is important for cortical waves and actin polymerization. We thus propose that the pool of FHDC1 that is involved in actin nucleation is regulated through its dynamic release from microtubule, likely at the shrinking ends (Figure 8E).

### FHDC1 knockdown cells display defects in cell polarity, locomotion and division

**FIGURE 9:**
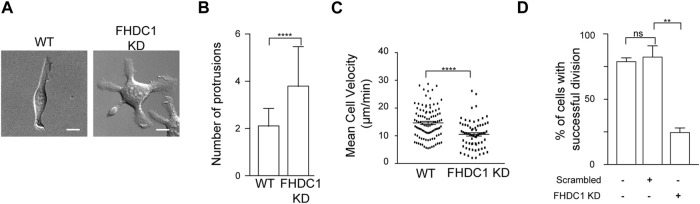
FHDC1 knockdown changes cell motility and impairs cell division. **(A)** FHDC1 knockdown cells present increased numbers of cell protrusions. Representative micrographs of wild type cell and cell transfected with FHDC1 shRNA imaged by DIC. Scale bar: 10 μm. **(B)** Quantification for number of cell protrusions between wild type cells and FHDC1 knockdown cells *(n* = 51 cells each for wild type and FHDC1 KD). **(C)** Quantification of cell velocity between wild type (*n* = 102 cells) and FHDC1 knockdown cells (*n* = 69 cells). **(D)** Quantification of percentage of cells with successful cell division between wild type cells (*n* = 194 cells; 3 independent experiments) and FHDC1 knockdown cells (*n* = 149 cells; 3 independent experiments).

To assess the physiological impact of perturbing FHDC1, we next characterized the cellular defects associated with FHDC1 depletion using shRNA. Many phenotypic defects could be found. One significant phenotype observed in FHDC1 knockdown cells was an increase in the number of cell protrusions (Figure 9A). Wild type cells generally exhibited two cell protrusions on average (2.2 ± 0.1 protrusions, 51 cells). However, upon FHDC1 knockdown, the number of observed protrusions significantly increased, with some cells displaying up to eight protrusions observed (3.8 ± 0.2 protrusions, 51 cells) (Figure 9B). This elevation suggests a reduced ability of FHDC1 knockdown cells to establish cell polarity. Furthermore, cell locomotion was impaired as indicated by a decrease in cell velocity from 14.7 ± 0.6 μm/min in wild type cells to 10.5 ± 0.6 μm/min in FHDC1 knockdown cells (*p* < 0.0001, Student’s t-test) (Figure 9C). FHDC1 knockdown also resulted in a significant reduction in the number of successful cell division events. In wild type cells, 80.1% ± 2.9% of the cells successful underwent division within a 24 h period (*n* = 194 cells; 3 independent experiments). Cells transfected with scrambled shRNA exhibited a comparable rate of successful division (83.7% ± 8.7%, *n* = 93 cells, 2 independent experiments). In contrast, cells transfected with FHDC1 shRNA demonstrated a substantial decrease, with only 25.5% ± 3.5% of the cells undergoing successful division (*n* = 149 cells, 3 independent experiments) (*p* = 0.0051, Student’s t-test) (Figure 9D). These results suggest that FHDC1 broadly impact cell function, likely due to its role in dynamically coordinating actin and microtubule cytoskeletons.

## Discussion

In this paper, we have demonstrated that formins play a crucial role in regulating actin wave propagation in mast cells. Recent studies have also highlighted the importance of formins in actin waves in *Dictyostelium* cells (Jasnin et al., 2016; Ecke et al., 2020) and *Xenopus* oocyte (Michaud et al., 2022). We have visualized travelling waves of the formin FHDC1 and microtubules coupled with cortical actin waves, which have not been previously observed. These observations allowed us to identify microtubule depolymerization and microtubule-binding formin protein FHDC1 as key regulators of actin waves.

The crosstalk of actin and microtubule dynamics could be regulated through various mechanisms (Dogterom and Koenderink, 2018). One mode of regulation involves direct physical crosslinking of actin and microtubule. Protein such as tau and spectraplakins are examples that possess both actin and microtubule-binding domains that allow them to directly link and align actin filaments with microtubules (Wu et al., 2008; Fontela et al., 2017). This physical interaction promotes co-alignment and coordination between the two cytoskeletal systems. Another form of crosstalk involves competition between actin and microtubules for binding to common interacting proteins (Henty-Ridilla et al., 2016). There are proteins that may have the ability to interact with both actin and microtubules and act as shared regulators. The binding of these proteins to either actin or microtubules can influence the dynamics of the respective cytoskeletal network, thereby indirectly affecting the dynamics of the other network. Additionally, actin and microtubules can share a common upstream regulator that coordinate their dynamics. RhoGTPases, such as Rho, Cdc42, and Rac are key regulators of both actin polymerization and microtubule dynamics. They can activate signaling pathways that mediate actin stress fiber formation or facilitate microtubule stabilization and organization (Waterman-Storer et al., 1999; Fukata et al., 2002; Krendel et al., 2002; Graessl et al., 2017).

There is growing evidence indicating that formins are prominent regulators of both actin and microtubules (Chesarone and Goode, 2010; Bartolini and Gunderson, 2011). Some formins, such as mDia1, mDia2 and mDia3, bind to microtubule using their FH domains, which are primarily involved in actin polymerization (Palazzo et al., 2001; Bartolini et al., 2008; Gaillard et al., 2011; Daou et al., 2014). Other formins, such as FMN1 and FHDC1, have separate microtubule-binding domains that allow them to associate with microtubules (Zhou et al., 2006; Young et al., 2008). Additionally, formins like DAAM1 may use a combination of FH domains and microtubule-binding domains to interact with microtubules (Szikora et al., 2017). Formins can bind to microtubules at different sites, such as along the entire microtubules (FHDC1, FMN2) (Young et al., 2008; Kwon et al., 2011) or accumulate as puncta along the sides or ends of microtubules (mDia1) (Henty-Ridilla et al., 2016). Furthermore, formins have been shown to modulate post-translational modifications of microtubules, such as detyrosination (Andrés-Delgado et al., 2012) and acetylation (Thurston et al., 2012; Fernández and Miguel, 2018). However, the functional implications of these mechanistic variances in formin-microtubule interactions are not yet fully understood.

In our study, we found that at least three formins expressed in RBL cells could have the potential to be involved in actin waves, although some may only participate in a subset of waves. This highlights the complexity of cortical dynamics, but the importance of formins in actin wave regulation is likely general. In *Dictyostelium* cells, inhibiting formins has been shown to suppress localized actin accumulation on perforations that are initiated by wave propagation (Jasnin et al., 2016). We focused on FHDC1 due to its robust involvement in actin waves and co-localization with microtubules, which distinguishes it from the other formins. FHDC1 exhibits a significant phase shift ahead of actin, suggesting that its actin nucleation function is likely inhibited when associated with microtubules. Microtubule binding can inhibit the actin nucleation activity of some formins including mDia1 and mDia2 (Gaillard et al., 2011; Bartolini et al., 2012). On the other hand, mutant lacking microtubule binding also fail to rescue actin wave formation, indicating the cytosolic FHDC1 could not function properly, and microtubule binding is important. Together with the observation that disrupting microtubules dynamics using the microtubule-stabilizing agent taxol led to the complete cessation of traveling waves and the correlation between actin assembly and phase of microtubule depolymerization but not phase of assembly, we propose that the microtubule shrinking ends regulate the propagation of actin nucleation, potentially through the local release and activation of FHDC1. Other possibilities could be that taxol has other signaling effects that are unrelated to FHDC1, or that FHDC1 regulates microtubule’s dynamic instability but its local release from the shrinking end may not be necessary. Microtubule at cell cortex could also associate with ER-PM (Endoplasmic Reticulum-Plasma Membrane) contact sites. Membrane contact can negatively regulate plasma membrane lipid signaling (Xiong et al., 2022). Thus, dissociating the contact site can have a positive effect on level of signaling lipids such as phosphoinositides which in turn regulates actin nucleation. However, these scenarios could not readily explain why the microtubule-binding segments of FHDC1 lacking actin nucleation activity (FHDC1 501-1143) completely failed to rescue wave propagation.

While there has been extensive characterization of proteins binding to the microtubule growing ends, our understanding of microtubule shrinking ends and their roles as potential sites of regulation remains limited. A prominent example is how kinetochore proteins have to remain attached to the shrinking ends of microtubules to translate forces generated from microtubule depolymerization to chromosome separation (Koshland et al., 1988; Westermann et al., 2006; Alushin et al., 2010; Volkov et al., 2018). Recent studies also showed that spastin, a microtubule-severing protein, can accumulate on shrinking ends of microtubule (Kuo et al., 2019; Al-Hiyasat et al., 2023). It is therefore an interesting possibility that FHDC1 may convert the forces of microtubule depolymerization to enhance the activity of actin nucleation. Consistent with this, formins are known to be mechanosensitive (Jégou et al., 2013; Yu et al., 2017, 2018; Cao et al., 2018). The possibility of antagonism between actin and microtubule, mediated by shrinking microtubules, introduces an additional layer of complexity on the crosstalk of actin and microtubule, that is essential for diverse cell structure and functions (Waterman-Storer et al., 1999; Wu et al., 2008; Bement et al., 2015; Ning et al., 2016; Winans et al., 2016; Wu and Bezanilla, 2018).

## Materials and methods

### Cell culture, transfection and drug treatments

Rat Basophilic Leukemia (RBL-2H3) cells were cultured as monolayer in MEM growth medium (Life Technologies, Carlsbad, CA) supplemented with 20% heat-inactivated Fetal Bovine Serum (Sigma-Aldrich, St Louis, MO). The cells were harvested with TrypLE Express (Life Technologies, Carlsbad, CA) 3 days after passage. Transient transfections were performed on cells in suspension by subjecting them to two pulses at 1200 mV for 20 ms using Neon Transfection Electroporator (Life Technologies, Carlsbad, CA). After transfection, cells were plated in 35 mm glass-bottom Petri dishes (*In vitro* scientific and MatTek) at sub-confluent densities and sensitized with anti-DNP IgE (Life Technologies, Carlsbad, CA) at 0.5 μg/mL. The cells were maintained overnight in a humidified incubator at 37°C. For experiments involving the use of inhibitors, the inhibitors were diluted from stock solutions and added to the cells at the following final concentrations: CK-666 (50 μM, Sigma-Aldrich, St Louis, MO), SMIFH2 (100 μM, Sigma-Aldrich, St Louis, MO), Taxol (100 nM, Sigma-Aldrich, St Louis, MO), Nocodazole (10 μM, Sigma-Aldrich, St Louis, MO).

To achieve targeted FHDC1 knockdowns, a combination of four unique 29mer pRFP-C-RS-FHDC1 short hairpin RNA (shRNA) constructs (TF705017A, B, C, D, OriGene Technologies Inc, Rockville, MD) were introduced into the cells. For controls, scrambled shRNA (TR30012, OriGene Technologies Inc, Rockville, MD) was used instead. Prior to imaging, the cells were incubated at 37°C in a humidified CO_2_ incubator for 48 h to allow for gene silencing and ensure maximum clearance of endogenous FHDC1. The selection of FHDC1 knockdown cells was based on the expression of turboRFP. To rescue the FHDC1 knockdown phenotype, shRNA-resistant constructs carrying human FHDC1 sequences (GFP-FHDC1, GFP-FHDC1(1-500) and GFP- FHDC1(501-1143) were co-transfected with the FHDC1 shRNAs. Additionally, iRFP-CBD was used as a readout for waves. After transfection, cells were incubated at 37°C in a humidified CO_2_ incubator for 48 h to allow for gene silencing and the expression of human FHDC1 before imaging.

### Molecular cloning and plasmids

To generate iRFP-N-Wasp (Wu Lab Plasmid ID: A414), the iRFP fluorescent tag was subcloned into the pEGFP-C1 backbone vector, replacing the EGFP, using kpnI site. The N-Wasp sequence was then subcloned from GFP-N-Wasp (Wu Lab Plasmid ID: A41) into the modified pEGFP-C1 vector. For construction of mCherry-FBP17 (Wu Lab Plasmid ID: F011d), the FBP17 was subcloned using XhoI and EcoRI sites from FBP17-EGFP (Wu Lab Plasmid ID: F011) into pmCherry-C1 backbone vector. Truncated GFP-FHDC1 1-500 (Wu Lab Plasmid ID: A104b) and GFP-FHDC1 501-1143 (Wu Lab Plasmid ID: A104c) mutant were generated by subcloning the respective sequences into pEGFP-C1 vector using the KpnI and BamHI restriction sites. The source of these constructs was the mCherry-FHDC1 (Wu Lab Plasmid ID: A103). Constructs for the following proteins were obtained as kind gifts: LifeAct-mRuby (Wu Lab Plasmid ID: A12) from Dr Roland Wedlich-Soldner (Max Planck Institute of Biochemistry, Martinsried, Germany); mEos2-Actin-7 (Wu Lab Plasmid ID: A04a) from Dr. Michael W. Davidson (Florida State University, Tallahassee, FL); GFP-α-tubulin (Wu Lab Plasmid ID: U01) from Dr Yih-Cherng Liou (National University of Singapore, Singapore); GFP-Arp3 (Wu Lab Plasmid ID: A31) from Dr Dorothy Schafer (University of Massachusetts Medical School, Worcester, MA); FBP17-EGFP and mCherry-actin from Dr Pietro De Camilli; GFP-mDia1 (Wu Lab Plasmid ID: A93), GFP-mDia3 (Wu Lab Plasmid ID: A99), FMNL2-GFP (Wu Lab Plasmid ID: A91), DAMM1-GFP (Wu Lab Plasmid ID: A92) and FHOD1-GFP (Wu Lab Plasmid ID: A94) from Dr Alexander Bershadsky (Mechanobiology Institute, Singapore); FMN1-GFP (Wu Lab Plasmid ID: A101) from Dr Michael Sheetz (Mechanobiology Institute, Singapore); GFP-FMN2 (Wu Lab Plasmid ID: A102) from Dr Sonia Rocha (University of Dundee, Dundee, Scotland, UK); FMNL1-GFP (Wu Lab Plasmid ID: A98) from Dr Michael Rosen (UT Southwestern Medical Center, Dallas, TX); mCherry-FHDC1(Wu Lab Plasmid ID: A103) from Dr John Copeland (University of Ottawa, Ottawa, Ontario, Canada).

### Microscopy

Before imaging, the medium in the dishes was replaced with Tyrodes’s imaging buffer (20 mM HEPES (pH 7.4), 135 mM NaCl, 5.0mM KCl, 1.8 mM CaCl_2_, 1.0 mM MgCl_2_ and 5.6 mM glucose). The cells were then transferred to a heated microscope stage (Live Cell Instrument, Seoul, South Korea) maintained at 37°C throughout the experiments. For TIRFM imaging of live cell cortical dynamics, a Nikon Ti-E inverted microscope (Nikon, Shinagawa, Tokyo) equipped with a perfect focus system to prevent focus drift was used. The microscope is also equipped with an iLAS2 motorized TIRF illuminator (Roper Scientific, Evry Cedex, France) and either an Evolve 512 EMCCD camera (Photometrics, Tucson, AZ) (16 bit, pixel size 16 μm) or Prime95b sCMOS camera (Photometrics, Tucson, AZ) (16 bit, pixel size 11 μm). Objective lenses from Nikon’s CFI Apochromat TIRF Series (100xH N.A. 1.49 Oil; 60xH N.A. 1.49 Oil) were used for image acuisition. Multi-channel imaging of samples was achieved by sequential exciting the samples with 491 nm (100 mW), 561 nm (100 mW) and 642 nm (100 mW) lasers, reflected from a quad-bandpass dichroic mirror (Di01- R405/488/561/635, Semrock, Rochester, NY) located on a Ludl emission filter wheel (Carl Zeiss AG, Oberkochen, Germany). The microscope was controlled using MetaMorph software (Version 7.8.6.0) (Molecular Devices, LLC, Suunyvale, CA). During image acquisition, the samples were maintained at 37°C using an on-stage incubator system (Live Cell Instrument, Seoul, South Korea). Prior to or during imaging, stimulation was performed by adding 80 ng/mL of DNP-BSA, a multivalent antigen that stimulates an antigen response. Movies were acquired following 15–90 min after stimulation. To ensure cell viability during long term imaging lasting more than 1 hour, the spent media was replaced with fresh media. Additionally, 5% humidified CO_2_ was maintained. For photo-conversion of mEos2-Actin, the 405 nm laser was set to its maximum power and pulsed on a single punctum for 20 ms. The photo-conversion process was controlled using the ‘On Fly’ module of the iLAS2 controller. To perform Fluorescence Recovery After Photobleaching (FRAP) on mCherry-FHDC1 and ensconsin-GFP treated with taxol and nocodazole, a region of interest (ROI) measuring 7 × 6 μm was selected, and photobleaching was carried out using the 561 nm laser set to maximum power. The FRAP experiments were controlled using the ‘On-Fly’ module of the iLAS2 controller.

To study the functional effects of FHDC1 knockdown on cell division and locomotion, wide-field epi-fluorescence and DIC microscopy were used in tandem. Image acquisition was performed using a Nikon Ti-E inverted microscope (Shinagawa, Tokyo, Japan). The microscope was equipped with an X-Cite 120LED microscope light source (Excelitas Technologies Corp, Waltham, MA, 370–700 nm) and an ORCA-Flash 4.0 V2 Digital CMOS camera C11440-22CU (Hamamatsu, 16 bit, pixel size 6.5 μm). All images were acquired using an objective len from Nikon’s CFI Plan Apochromat Lambda (λ) Series (40x N.A. 0.95). The microscope was controlled using the NIS-Elements AR 4.20 software (Nikon, Shinagawa, Tokyo). To ensure cell survival during image acquisition, the samples were maintained at 37°C with a 5% humidified CO_2_ environment.

### Protein purification

Truncating variants of FHDC1 were fused with an N-terminal GST-His6 tag in pNIC-GST vector using ligation-independent cloning (LIC) technology. Profilin-1 was fused with N-terminal 8xHis tag in a modified version of pET-21d (+) pSY5 vector. The respective bacterial expression vectors were transformed into E.coli BL21 (DE3) Rosetta T1R and cultured in Terrific Broth supplemented with 8 g/L glycerol. The cultures were incubated at 37°C with shaking at 200 rpm overnight and induced with 0.5 mM IPTG for protein expression. The induced cultures were further incubated overnight at 18°C. Cells were harvested and resuspended in lysis buffer (100 mM HEPES, 500 mM NaCl, 10 mM Imidazole, 10% glycerol, 0.5 mM TCEP, pH 8.0) supplemented with protease inhibitor cocktail set III, EDTA free (diluted 1000x in lysis buffer, Calbiochem, USA), and benzonase (Merck, USA) at a final concentration of 5 μL per liter of culture. The re-suspended cell pellet were sonicated using a Sonics Vibra-cell at 70% amplitude for 3 min on ice with a cycle of 3 s on/off. The lysate was clarified by centrifugation at 47,000 g, 4°C for 25 min. The supernatants were filtered through 1.2 μm syringe filters and loaded onto an AKTA Xpress system (GE Healthcare). The lysates were loaded on IMAC columns and. washed with binding buffer (20 mM HEPES, 500 mM NaCl, 25 mM Imidazole, 10% (v/v) glycerol, 0.5 mM TCEP, pH 7.5). Gradient elution was performed using elution buffer (20 mM HEPES, 500 mM NaCl, 500 mM Imidazole, 10% (v/v) glycerol, 0.5 mM TCEP, pH 7.5). The eluted proteins were collected, stored in sample loops on the system and injected into a Gel Filtration column (HiLoad 16/60 Superdex 200 prep grade, GE Healthcare). The protein sample was concentrated using Vivaspin 20 filter concentrators (VivaScience, USA), aliquoted into smaller fractions, flash frozen in liquid nitrogen and stored at −80°C.

### Pyrene-actin polymerization assays

Pyrene-actin polymerization assays were performed following the protocol described in previous studies (Miao et al., 2013; Sun et al., 2018). Rabbit skeletal muscle actin was purified from rabbit muscle acetone powder (Pel-Freez, USA). Pyrene-labeled actin was obtained from Cytoskeleton Inc. Monomeric actin at a concentration of 2 µM was mixed with 5% pyrene-labeled actin in the actin polymerization reactions. The fluorescence of pyrene was monitored using a fluorescence spectrophotometer (Cytation 5, BioTek, USA). The obtained data were analyzed and plotted using Origin software (Originlab Corporation, USA).

### RNA sequencing (RNA-Seq) and analysis

Total RNA extraction was carried out using the GeneJET RNA Purification Kit (ThermoFisher Scientific, Waltham, MA) following the manufacturers’ protocol. The purity of the extracted RNA was assessed using the Nanodrop 2000c Spectrophotometer (ThermoFisher Scientific, Waltham, MA). Subsequently, the RNA samples were sent to the RNA sequencing facility at Beijing Genomic Institute (Hong Kong) for cDNA library preparation and sequencing. For gene expression analysis, the numbers of reads uniquely mapped to the specific genes and the overall number of uniquely mapped reads in the sample were determined. The gene expression levels were calculated using the RPKM (Reads Per Kilobase per Million mapped reads) method. Raw data for the RNA-Seq used in this paper has been deposited to Dyrad (https://doi.org/doi:10.5061/dryad.866t1g1wj) and is publicly accessible.

### Image analysis

Post-acquisition image analyses were performed using either Fiji (Schindelin et al., 2012) or MATLAB (The MathWorks, Inc., Natick, MA). Kymographs were generated using the “Reslice” tool. Depending on intensity of the fluorescent probes, an “average” projection filters (average of 10 frames) and background subtractions may have been applied to enhance presentation. For consistency, the same processing was applied across all channels for kymographs generated from multi-color imaging. Additionally, the processing parameters were applied identically across different conditions that were directly compared to each other. Montages were generated using the “Make Montage” tool. For intensity profiles, a region of interest with a size of 20 × 20 pixels was used for all movies. The intensity data points generated by Fiji and were normalized and plotted using MATLAB. Time projection images were created by merging three sequential frames at equal intervals and applying pseudocolors. An “average profile” was generated by aligning multiple cycles of their intensity fluctuations according to their peaks, to illustrate phase differences between proteins in wave propagation. In the generated plots, solid lines represent the mean intensities, and shaded region represents the standard deviations of the intensities. The time delay between two probes was quantified by cross-correlation analysis. The code used to generate these plots have been previously reported and published on GitHub (https://github.com/min-wu-lab/mmo-analysis) (Tong et al., 2023). For determination of wave percentage displayed by each individual formin in Figure 3, we first grouped the cells with and without wave propagation visually through generated kymographs. Majority of the wave patterns are positive waves, where the intensities of formins are higher compared to the baseline. Cells displaying “negative” waves, defined by having a decrease in intensities compared to baseline levels, are grouped with cells not exhibiting wave propagation, as they could result from membrane fluctuations. We next performed fast Fourier transformations (FFTs) on the intensity profile, using the presence of distinct frequency peaks in the range of 0–40 s as a criterion. To assess the impact of FHDC1 knockdown on cell division, cells transfected with FHDC1 shRNA were imaged using DIC microscopy. The percentage of successful divisions was quantified by visually examining the images. Quantification of cell velocity was performed using the ‘Manual Tracking’ plugin in Fiji. The x and y coordinates of the center of the cell body was recorded and utilized to calculate the velocity of FHDC1 knockdown or control cells.

### Statistical analysis

All statistical analyses were conducted using Prism 7 software (GraphPad Software, Inc, La Jolla, CA). To compare control and perturbed samples, unpaired two-tailed Student’s t-tests were performed. For multiple comparisons in Figures 3D, 4M, a one-way ANOVA followed by Sidak’s multiple comparison *post hoc* test was performed. Unless otherwise stated, error bars in all data shown represent mean ± S.E.M.

## Data Availability

The original contributions presented in the study are publicly available. This data can be found here: https://datadryad.org/stash/share/Z7pp-zH9pnOc_TadfflYp5MdVTtYywRqjT9GIg3lMsc.

## Supplementary material

SUPPLEMENTARY MOVIE S1 TIRFM movie of a cell co-expressing FBP17-EGFP and LifeAct-mRFPruby showing cortical travelling waves. FBP17 wave (cyan) is phase-shifted compared to that of LifeAct (magenta). The movie was acquired at 2 s interval and plays at 10 frames per second (20X real time). Scale bar: 10 μm.

SUPPLEMENTARY MOVIE S2 TIRFM movie of a cell co-expressing GFP-α-tubulin (left) and mCherry-FHDC1 (middle) showing travelling waves. α-tubulin waves (cyan) precisely overlap with FHDC1 waves (magenta) in the merged panel (right). The movie was acquired at 2 s interval and plays at 40 frames per second (80X real time). Scale bar: 10 μm.

SUPPLEMENTARY MOVIE S3 TIRFM movie of a cell co-expressing GFP-α-tubulin, mCherry-FHDC1 and iRFP-N-WASP showing travelling waves. GFP-α-tubulin (green) is colocalized to mCherry-FHDC1 (red) and phase-shifted compared to that of N-Wasp (blue). The movie was acquired at 1 s interval and plays at 20 frames per second (20X real time). Scale bar: 10 μm.

SUPPLEMENTARY MOVIE S4 TIRFM movie of a cell co-expressing GFP-EB1, mCherry- α-tubulin and iRFP-N-Wasp showing microtubule shrinkage at its plus ends. Microtubules shrinkage (red) happens upon the arrival of iRFP-N-Wasp wave (blue) at microtubule tips, labeled by GFP-EB1 (green). The movie was acquired at 1 s interval and plays at 20 frames per second (20X real time) for the whole cell view and plays at 10 frames per second (10X real time) for zoom-in view. Scale bar: 10 μm.
